# A Polysaccharide Isolated from *Dictyophora indusiata* Promotes Recovery from Antibiotic-Driven Intestinal Dysbiosis and Improves Gut Epithelial Barrier Function in a Mouse Model

**DOI:** 10.3390/nu10081003

**Published:** 2018-07-31

**Authors:** Sadia Kanwal, Thomson Patrick Joseph, Lawrence Owusu, Ren Xiaomeng, Li Meiqi, Xin Yi

**Affiliations:** 1 Department of Biotechnology, College of Basic Medical Sciences, Dalian Medical University, Dalian 116044, Liaoning, China; sadiakanwal490@gmail.com (S.K.); renxiaomeng319@gmail.com (R.X.); limeiqi07@gmail.com (L.M.); 2 Department of Microbiology, College of Basic Medical Sciences, Dalian Medical University, Dalian 116044, Liaoning, China; microbiologist02@gmail.com; 3 Department of Biochemistry and Biotechnology, Kwame Nkrumah University of Science and Technology (KNUST), PMB, UPO, Kumasi 00000, Ghana; dady_k_jrn@yahoo.com

**Keywords:** gut microbiota, broad-spectrum antibiotics, dysbiosis, *Dictyophora indusiata*, polysaccharide, restoration, immunology

## Abstract

Despite the tremendous biological activity of polysaccharides from the mushroom *Dictyophora indusiata*, its role in the restoration of gut microbiota has not yet been explored. The present study aimed to investigate whether *D. indusiata* polysaccharide (DIP) could modulate the recovery of gut microbiota composition and intestinal barrier function after broad-spectrum antibiotic-driven dysbiosis. Alteration and restoration in the microbial communities were elucidated by the Illumina MiSeq platform. Colon histology, expression of tight-junction associated proteins, and serum/tissue endotoxin and cytokine levels were evaluated. Two-week daily oral administration of clindamycin and metronidazole resulted in reduced bacterial diversity and richness, and perturbed the microbial flora at various taxonomic levels (altered Firmicutes/Bacteroidetes ratio and increased relative abundance of harmful flora (Proteobacteria, *Enterococcus*, and *Bacteroides*)), whereas DIP administration reversed the dysbiosis and increased beneficial flora, including Lactobacillaceae (lactic acid-producing bacteria), and Ruminococaceae (butyrate-producing bacteria). In addition, it resulted in the reduction of endotoxemia (through lipopolysaccharides (LPSs)) and pro-inflammatory cytokine (tumor necrosis factor alpha (TNF-α), interleukin 6 (IL-6), and interleukin 1β (IL-1β)) levels, with the increased expression of tight-junction associated proteins (claudin-1, occludin, and zonula occludens-1). These findings not only suggested a comprehensive understanding of the protective effects of a DIP in the restoration of gut microbiota but also highlighted its role in the enhancement of gut barrier integrity, reduction of inflammation and lowering of endotoxin levels in mice.

## 1. Introduction

The gut microbiota is the complex and highly dynamic community of microorganisms that colonize the gastrointestinal tract. It is now well recognized as a promising contributing factor in the determination of host health and physiology [1,2]. The undesirable changes in the composition of gut microbiota have been reported to be involved in a variety of human ailments, including inflammatory bowel diseases (IBD) [3,4,5,6], type 2 diabetes [7], atherosclerosis [8], chronic kidney disease [9], and mental illnesses [10,11,12,13]. Hence, the assessment of the microbiome of the healthy and diseased individual could provide some novel clues about the underlying role of gut microbiota influencing host health in terms of disease initiation and progression.

The elements that influence the gut microbiome include the alterations in diet, age, genetic background, use of antibiotics, and environmental factors [14,15,16]. The effect of antibiotics is a concern as they are extensively used for the treatment and prevention of bacterial infections; recent findings revealed that antibiotics can have an adverse impact on the host physiology, causing alterations in the intestinal flora, a condition known as ‘dysbiosis’ or ‘dysbacteriosis’ [17,18,19,20,21,22,23,24]. According to an estimate, in the United States, around 21% of antibiotics are prescribed with unclear indications for pediatric ambulatory visits, which partially include broad-spectrum antibiotics [24]. Broad-spectrum antibiotics can impact the overall abundance of bacterial composition causing a rapid decline in diversity, evenness, and taxonomic richness [25]. Several lines of evidence showed that treatment with broad-spectrum antibiotics like metronidazole, ampicillin, clindamycin, or vancomycin promote dysbiosis in in vivo models [26,27,28,29]. Moreover, recent findings have demonstrated the clear link between antibiotic exposure in early life and the increased risk of IBD development, and disturbances in intestinal homeostasis and epithelial barrier integrity in neonates [30,31,32]. Thus, an understanding of the factors that results in the alteration of gut microbiota and the potential strategies that can be used to restore the gut ecology is becoming increasingly important to clinicians and the induction of novel compounds and/or treatment strategy is of utmost importance. Emerging dietary strategies recommended for modulating the composition of the human gut microbiota include the use of probiotics, prebiotics, and polyphenols [33]. As far as prebiotics are concerned, they are the selectively fermented ingredients that confer beneficial effects on the host health by modulating the composition and/or the activity of the gut microbiota [34,35]. In addition, a fiber-rich diet not only affects the composition or activity of the microbiota but also provides physical benefits such as by increasing fecal bulking and laxation [36]. Another important feature of dietary fiber includes polysaccharides (prebiotics) that are not digestible by humans; however, they serve as an essential substrate for the microbial community inhabiting the distal gut, where they cause the degradation of dietary polysaccharides into short-chain fatty acids (SCFAs) that are absorbable by humans and also provide beneficial effects to host health [37,38].

Polysaccharides are polymeric carbohydrate molecules in which a large number of monosaccharides are mutually joined by glycosidic linkages [39]. Polysaccharides may be linear or branched and on the basis of the types of monomers present, they can be homo- or heteropolysaccharides [40]. The extraction, characterization and the biological activities of polysaccharides have been reported from several natural resources, where the role and contribution of edible and medicinal mushrooms cannot be overlooked [41,42,43,44,45,46,47]. One of such natural resource is the mushroom *Dictyophora indusiata* (Vent. ex Pers) Fisch (Chinese name Zhu Sun, meaning the bamboo mushroom), synonymously called *Phallus indusiatus*, that has been used as a medicinal mushroom since 618 AD in China [48]. This mushroom has a cosmopolitan distribution and has been consumed in a variety of folklore situations by ethnic groups [48,49]. Beside other components, polysaccharides isolated from the fruiting body of *D. indusiata* have been reported to show considerable bioactivities, such as immunostimulatory [50], anticancer [51], antioxidant [52], neuroprotective [53], and antiproliferative activities [54]. However, to the best of our knowledge, its effect on the gut microbial ecosystem and gut barrier function remain unknown.

Based on the evidence presented above and the limited number of investigations on the role of polysaccharides from natural resources in the restoration of antibiotic-induced gut dysbiosis, our research group aimed to address the hypothesis that polysaccharides (prebiotics) could potentially restore and/or induce favorable changes in the composition of gut microbiota and could also improve the host immunity. Therefore, in the present study, our prime focus was to investigate: (1) the impact of two different broad-spectrum antibiotics, i.e., clindamycin and metronidazole, on gut microbiota composition and intestinal barrier integrity, and (2) to explore the potency of crude polysaccharide from the mushroom *D. indusiata* (designated herein as DIP) in the alleviation and restoration of antibiotic-induced gut dysbiosis and barrier disruption and to determine the bacterial strains that may be associated with the health ameliorating properties. For this purpose, we determined the perturbation in the bacterial population in the fecal samples of BALB/c mice after antibiotic-induced dysbiosis and treatment with the crude polysaccharide fraction isolated from the mushroom *D. indusiata* (DIP), by 16S rRNA sequencing with the Illumina MiSeq platform. Moreover, histological analysis was done followed by western blotting and ELISA for barrier integrity-related protein expression (claudin-1, occludin, and zonula occludens-1 (ZO-1)) and pro-inflammatory-associated cytokine (tumor necrosis factor alpha (TNF-α), interleukin 6 (IL-6), and interleukin 1β (IL-1β)) analysis, respectively. Our findings indicated that the crude polysaccharide (DIP) from *D. indusiata* resulted in the reversion of antibiotic-induced dysbiosis and intestinal barrier disruption. In addition, it has also exerted a protective effect by increasing the population of health-promoting bacteria in the mice model used.

## 2. Materials and Methods

### 2.1. Experimental Animals and Housing

Specific-pathogen-free (SPF) level inbred male BALB/c mice aged 6–8 weeks old and weighing 18 ± 2 g were used in this study that were approved by the Animal Care and Research Ethics Committee of Dalian Medical University (Approval Number: SYXK 2016–2018). All the animals were randomly divided and accommodated according to the treatment group in separate cages and maintained in conventional conditions in an environmentally controlled room (20–22 °C temperature and 55 ± 5% relative humidity with 12–12 h light/dark cycle), with standard commercial chow (Jiangsu Medison Biomedical Co., Ltd., Yangzhou, Jiangsu Province, China) and water ad libitum. Prior to the experimentation, all the animals used in the study were acclimated for a period of one week in quarantine. All experimental procedures were approved by the ethics committee of Dalian Medical University.

### 2.2. Mushroom, Antibiotics, Antibodies, Kits, and Reagents

The fruiting body of the mushroom *Dictyophora indusiata* was obtained from Anhui Joy Lok Food Co., Ltd., Ningde, Fujian Province, China. The broad-spectrum antibiotics clindamycin hydrochloride and metronidazole were from Tian Fang Pharmaceutical Co., Ltd. (Zhumadian, China) and Changchun Wan de Pharmaceutical Co., Ltd. (Changchun, China) respectively. The DNA extraction kit (QIAamp DNA Stool Mini Kit) and gel purification kit (Agencourt AMPure XP 60 mL Kit) were from Qiagen (Hilden, Germany) and Beckman Coulter (Brea, CA, USA), respectively. The primary antibodies (β-actin, claudin-1, occludin, and zonula occludens-1 (ZO-1)), secondary antibodies, and the Radioimmunoprecipitation assay (RIPA) buffer were from Proteintech (Wuhan, China). The Mucin-2 (MUC2) primary antibody was from Boster Biological Technology, Wuhan, China. The horseradish peroxidase-conjugated secondary antibody and DAB substrate chromogen system were from ZSGB-BIO (Beijing, China). The bicinchoninic acid (BCA) protein assay kit was from Pierce Rockford, IL, USA. The Limulus amebocyte lysate (LAL) assay kit was obtained from Chinese horseshoe crab reagent manufactory Co., Ltd. (Xiamen, China). The ELISA kits were purchased from Shang Hai Lengton Bioscience Co, Ltd., Shanghai, China. All the other chemicals used in this study were of analytical grade and purchased from the standard commercial sources.

### 2.3. Extraction of Crude Polysaccharides (DIP) from the Fruiting Body of Dictyophora indusiata

The extraction of crude polysaccharide from the fruiting body of the mushroom *D. indusiata* was done following the procedure reported previously [55]. Briefly, the fruiting bodies of *D. indusiata* were dried in a hot air-drying oven at 45 °C and crushed into powder using a tissue triturator. The powder of fruiting body was extracted by using boiling water and the collected supernatant was then concentrated at 60 °C using a rotary evaporator. After that, the concentrated supernatant was deproteinated several times by the Sevag method [56] and the protein content was determined by the bicinchoninic acid (BCA) protein assay kit (Pierce, Rockford, IL, USA) following the manufacturer’s instructions. The resulting solution was ethanol precipitated, dried using vacuum freeze drying and collected as a crude polysaccharide (DIP).

### 2.4. Analysis of the Sugar Content and Monosaccharide Composition

The carbohydrate content in the crude polysaccharide (DIP) was determined by the phenol-H_2_SO_4_ method [57] and the monosaccharide composition was analyzed by using high-performance liquid chromatography (HPLC) technique [58].

### 2.5. Experimental Design, Antibiotic and Polysaccharide Treatment

**Figure 1:**
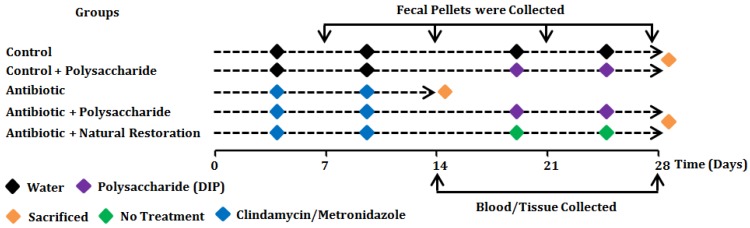
Experimental design of the study. Experimental plan outline where a vehicle or control group (Ctrl, *n* = 6) was gavaged with distilled water from days 0 to 28. Control + polysaccharide group (Ctrl + DIP, *n* = 6) received distilled water from days 0 to 14 followed by polysaccharide (DIP) treatment from days 15 to 28. Antibiotic groups (Clin/Met, *n* = 6 per group) received antibiotics, either clindamycin (0.2 mg/0.2 mL) or metronidazole (3.5 mg/0.2 mL), from days 0 to 14 and were sacrificed on day 15. Antibiotic + polysaccharide groups (Clin + DIP/Met + DIP, *n* = 6 per group) received antibiotics (clindamycin (0.2 mg/0.2 mL) or metronidazole (3.5 mg/0.2 mL)) from days 0 to 14 followed by DIP treatment from days 15 to 28. Antibiotic + natural restoration groups (Clin + NR/Met + NR, *n* = 6 per group) received antibiotics from days 0–14 followed by no treatment from days 15 to 28 to observe natural restoration.

The detailed experimental plan is presented in the Figure 1. In this study groups/treatments or a combination thereof were abbreviated as follows: Control, Ctrl; Clindamycin, Clin; Metronidazole, Met; Natural restoration, NR; and Crude Polysaccharide from *D. indusiata*, DIP. After the acclimation period of one week in quarantine, mice were treated daily with two broad-spectrum antibiotics i.e., clindamycin (0.2 mg/0.2 mL) and metronidazole (3.5 mg/0.2 mL) or vehicle (distilled water 0.2 mL) by oral gavage for 14 days. After this modelling period, antibiotic-treated groups (Clin and Met) were sacrificed on the 15th day by cervical dislocation, whereas the polysaccharide-treated groups (Clin + DIP and Met + DIP) received DIP (0.2 mg/0.2 mL) via oral gavage and the natural restoration groups (Clin + NR and Met + NR) did not received any treatment in order to observe natural restoration however, given normal diet for another 14 days after antibiotic treatment. Throughout the experimentation, the weight of each mouse was recorded twice weekly, and feces were collected on the 7th, 14th, 21th, and 28th days, transferred into sterile eppendorf (EP) tubes, immediately frozen in liquid nitrogen, and stored at −80 °C. The fecal samples collected were divided into two parts for Polymerase Chain Reaction-Denaturing gradient gel electrophoresis (PCR-DGGE) analysis and 16S rRNA Illumina sequencing. At the end of the experiment, blood and tissue samples were collected; sera were kept at −80 °C while the tissue samples were frozen immediately in liquid nitrogen prior to further analysis. Colon tissues were fixed in 10% formalin for histological analysis.

### 2.6. DNA Extraction and 16S rRNA Gene Amplification (Illumina MiSeq Sequencing)

DNA was extracted from fecal content according to the manufacturer’s instructions of QIAamp DNA Stool Mini Kit (Qiagen, Hilden, Germany). The quality and quantity of the genomic DNA were determined by electrophoresis and spectrophotometrically using BioPhotometer plus (NanoVue, USA) respectively. The 16S rRNA gene V4 region was amplified from genomic DNA using the universal bacterial primers: (515F, 5′-GTGCCAGCMGCCGCGGTAA-3′, and 806R, 5′-GGACTACHVGGGTWTCTAAT-3′) with the following protocol: Pre-denaturation at 98 °C for 30 s, denaturation at 98 °C for 15 s, annealing at 58 °C for 30 s, followed by elongation at 72 °C for 15 s, and the final extension at 72 °C for 1 min. The PCR products were separated and detected on a 1% agarose gel, then purified by Agencourt AMPure XP 60 mL Kit (Beckman Coulter, Brea, CA, USA) and quantified with Qubit dsDNA HS Assay Kit (Life Technologies, Carlsbad, CA, USA) according to the manufacturer’s instructions. The amplicons were normalized, pooled and sequenced on the Illumina Hiseq4000 sequencer (2 × 150 bp paired-end).

Libraries were prepared using Library Quant Kit Illumina GA revised primer-SYBR Fast Universal (KAPA, Wilmington, MA, USA) and sequenced for 600 cycles on an Illumina MiSeq using the MiSeq Reagent Kit (Illumina, San Diego, CA, USA). Quality sequences were acquired by removing low-quality reads and chimeras identified using Chimera.uchime. Taxonomic assignment of individual datasets was performed using the SILVA128. Operational taxonomic units (OTUs) were generated using Vsearch v1.11.1 with a dissimilarity cutoff of 0.03. Alpha diversity was calculated with Quantitative Insights into Microbial Ecology (QIIME) and beta diversity was analyzed using QIIME with the matrix of the Bray–Curtis index to measure similarity in microbial composition between samples.

### 2.7. Biochemical Analysis

Blood samples were centrifuged at 3000× *g* for 10 min and serum is separated to evaluate serum endotoxin (lipopolysaccharide, LPS) levels using a commercial kit based on the guidelines provided by the supplier (LAL, Limulus amoebocyte lysate assay; Chinese Horseshoe Crab Reagent Manufactory Co., Ltd. Xiamen, China). Colon tissue samples were homogenized with a certain amount of Phosphate Buffered Saline (PBS) (pH 7.4) using mortar and pestle. The resulting homogenates were centrifuged at 3000 rpm for 20 min and the supernatant was collected. The levels of the pro-inflammatory cytokines (tumor necrosis factor alpha (TNF-α), interleukin 6 (IL-6), and interleukin 1β (IL-1β)) in the serum and tissue were determined using a mouse ELISA kit (Shang Hai Lengton Bioscience Co., Ltd. Shanghai, China, according to the manufacturer’s instructions.

### 2.8. *Histological Analysis*

The distal colon of the mice was harvested and fixed in 4% paraformaldehyde for 24 h, dehydrated using gradient ethanol and vitrificated by xylene and embedded in paraffin. Then, 3 μm thick sections were prepared by microtomes (Thermo, Waltham, MA, USA). Sections were deparaffinized in xylene and rehydrated through decreasing grades of ethanol, followed by staining with hematoxylin and eosin (H&E), then gradient alcohol and xylene dehydration. Images were obtained using a microscope (Leica Microsystems, Wetzlar, Germany). The histological changes were evaluated in a blinded manner by an independent researcher.

### 2.9. Immunohistochemical Analysis for Mucin-2 (MUC2)

Mucin-2 expression levels in the gut were analyzed using Immunohistochemistry. Briefly, paraffin-embedded gut tissue sections were cut into 3 μm widths, deparaffinized in xylene, and rehydrated with graded ethanol. Tissues were incubated with 3% H_2_O_2_ (Sangon Biotech Co. Ltd. Shanghai, China) for 10 min. Antigen retrieval was performed by heating the tissue slides in antigen retrieval buffer (Na^+2^ EDTA, pH 8.0). The slides were incubated with primary antibody, rabbit-polyclonal anti-mouse MUC2 (catalog no. PB0156, 1:100) overnight at 4 °C. The slides were then permeated with a horseradish peroxidase-conjugated secondary antibody (catalog no. SP-9001) for 1 h at room temperature, washed with phosphate-buffered saline three times, stained with the 3,3-iaminobenzidine (DAB) substrate chromogen system (catalog no. ZLI-9018) for 1–5 min, and then counterstained with hematoxylin. Finally, the slides were mounted and observed under a light microscope at a magnification of ×20, Scale bar 100 µm. The histological alterations were examined in a blinded manner by an independent researcher.

### 2.10. Western Blotting

Total protein was extracted from the colon with RIPA buffer (Proteintech, Wuhan, China) supplemented with protease inhibitor cocktail (Transgene Biotech, Beijing, China) on ice for 30 min. After centrifugation at 14,000 rpm for 20 min at 4 °C, the protein content in the supernatant was determined using a BCA protein assay kit (Pierce, Rockford, IL, USA) according to the manufacturer’s instructions. Total protein lysates (20 µg) were fractionated on a 10% sodium dodecyl sulfate-polyacrylamide gel (SDS-PAGE) and transferred onto polyvinylidene difluoride (PVDF) membranes (Immobilon TM-P; Millipore, Massachusetts, USA). Membranes were blocked with blocking buffer (5% skimmed milk in TBS-T (20 mM Tris-HCl (pH 7.5), 150 mM NaCl, 0.1% Tween 20) for 1 h at room temperature in order to prevent non-specific antibody binding and then probed with following primary antibodies: β-actin (catalog no. 20536-1-AP, 1:2000), claudin-1 (catalog no. 13050-1-AP, 1:500), occludin (catalog no. 13409-1-AP, 1:2000) and ZO-1 (catalog no. 21773-1-AP, 1:500) overnight at 4 °C diluted with blocking buffer. After washing three times with TBS-T, membranes were then incubated at room temperature for 1 h with the horseradish peroxidase-conjugated secondary antibody: goat anti-rabbit Immunoglobulin G (IgG) (catalog no. SA00001-2, 1:6000)). Blots were then developed with WesternBright™ ECL substrate (Advansta, Inc., Menlo Park, CA, USA) and images were captured by ChemoDoc^TM^ XRS + Imager-Bio-Rad (Bio-Rad Laboratories, Inc., Hercules, CA, USA).

### 2.11. Statistical Analysis

All the statistical analysis was performed with GraphPad Prism 5.01 software (La Jolla, CA, USA). Statistical significance was determined by using one-way analysis of variance (ANOVA) followed by Tukey’s multiple comparison test and *p* < 0.05 was considered to be statistically significant.

## 3. Results

### 3.1. Extraction and Monosaccharide Composition Analysis of Crude Polysaccharide DIP from the Mushroom D. indusiata

The crude polysaccharide was extracted from the mushroom *D. indusiata* by hot water extraction and ethanol precipitation method. The concentration of crude polysaccharide (DIP) was determined to be 12.76 mg/mL using D-glucose as standard. The yield, total sugar content, protein content, and the monosaccharide composition of the crude polysaccharide (DIP) obtained is presented in Table 1.

### 3.2. Experimental Strategy and the Effect of DIP on the Body Weight of Antibiotic-Treated Mice

In our study, dysbiosis was induced in BALB/c mice using the two broad-spectrum antibiotics clindamycin and metronidazole, and restoration was achieved by a DIP fraction with the indicated concentration and time as illustrated in the material and methods section and Figure 1.

**Figure 2:**
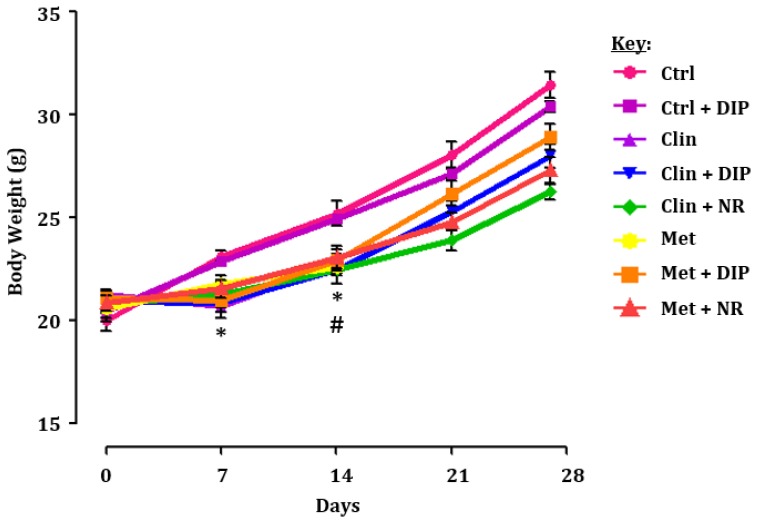
Body weight measurement after *Dictyophora indusiata* polysaccharide (DIP) treatment in an antibiotic-treated mouse model at different time points. Mice receiving clindamycin (0.2 mg/0.2 mL) or metronidazole (3.5 mg/0.2 mL) via oral gavage had a significantly lower body weight in comparison with the control (Ctrl) group (one-way analysis of variance (ANOVA) followed by Tukey’s multiple comparison test). Data presented here is the mean ± standard error of mean (SEM) (*n* = 6). * *p* < 0.05 clindamycin-treated group compared to control group, # *p* < 0.05 metronidazole-treated group compared to control group.

We assessed the body weight of each mice during the treatment, which revealed a significant reduction in the body weight of the clindamycin-treated group (Clin) at 1 and 2 weeks post-antibiotic treatment in comparison with the control group (Ctrl) (* *p* < 0.05), while in metronidazole-treated group (Met) a significant reduction was observed after 2 weeks of antibiotic treatment as compared to the control group (Ctrl) (# *p* < 0.05). However, both antibiotic-treated groups given DIP treatment (Clin + DIP/Met + DIP) after 14 days showed an increase in the body weight as compared to natural restored groups (Clin + NR/Met + NR) (Figure 2).

### 3.3. Alpha Diversity Indices Were Restored by DIP Treatment in Antibiotic-Treated Mice

Preliminary status, dysbiosis, and restoration were assessed with the conventional PCR-DGGE fingerprint analysis, which revealed the dynamic changes and reduction in the bacterial diversity in both the antibiotic-treated groups (Clin/Met) in comparison with the control group (Ctrl) and DIP-treated groups (Clin + DIP/Met + DIP) (Supplementary Figure S1). In contrast to the decreasing trend of the microbial population in antibiotic-treated groups (Clin/Met), some bands were denser and thicker compared to the control group (Ctrl). Moreover, several bands that became weaker or even disappeared in the antibiotic-treated groups (Clin/Met), reappeared or became stronger in the groups that were treated with a DIP after respective antibiotic treatment (Clin + DIP/Met + DIP). On the other hand, naturally restored groups (Clin + NR/Met + NR) failed to recover all the bacterial flora as observed in the control and DIP-treated groups. (Supplementary Figure S1). The results of PCR-DGGE indicated that the antibiotic treatment (Clin/Met) resulted in the reduction of bacterial diversity that was restored more efficiently upon treatment with DIP (Clin + DIP/Met + DIP) in comparison with the naturally restored groups (Clin + NR/Met + NR).

**Figure 3:**
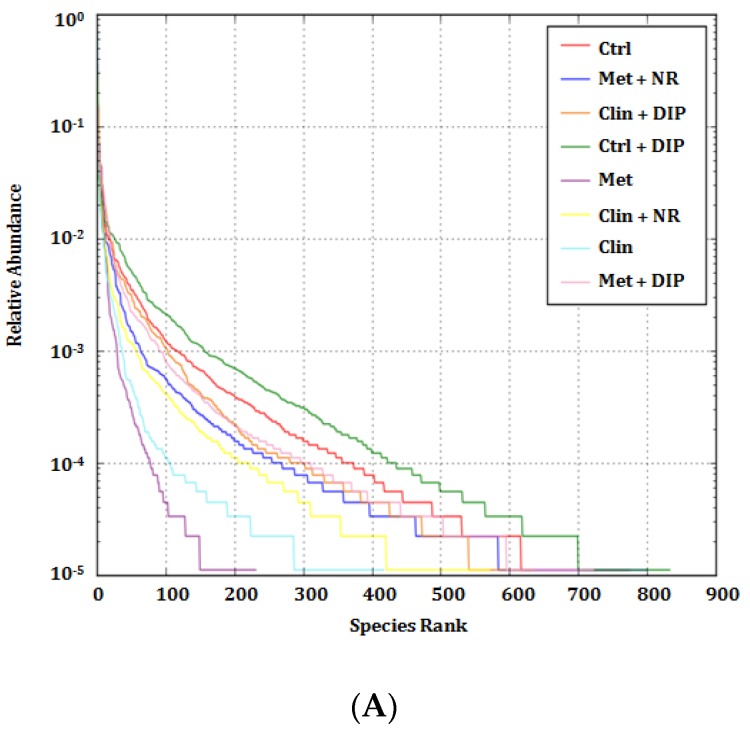

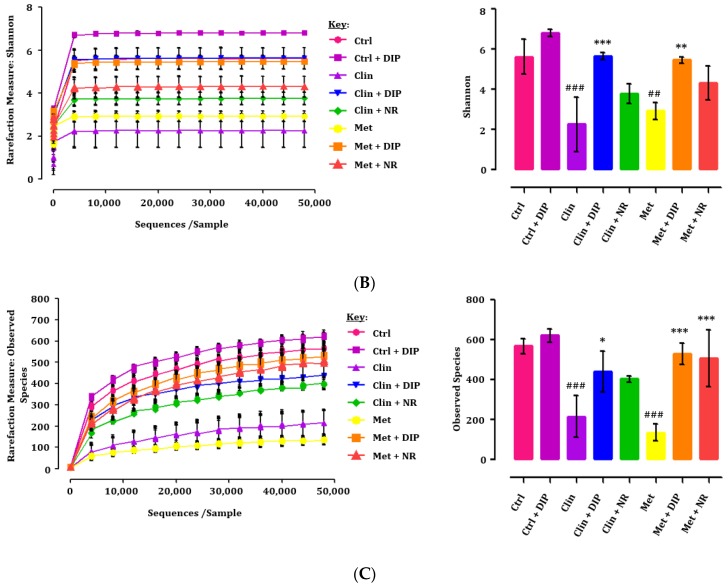
Rank abundance and rarefaction curves. (**A**) Rank abundance curve depicting the species richness and evenness. The *x*- and *y*-axis indicates the number of operational taxonomic units (OTUs) according to the relative abundance and species evenness respectively. The higher the species richness, the larger the curve on the *x*-axis will be, and the smoother the curve, the more even the species distribution will be. (**B**) Rarefaction curves representing species abundance and diversity. Left: Shannon index value showing the microbial diversity and richness for each treatment group. Right: bar graph of the Shannon index values at maximum OTUs, where ## *p* < 0.01 and ### *p* < 0.001 compared to the control group (Ctrl), and ** *p* < 0.01 and *** *p* < 0.001, compared to the antibiotic-treated groups (Clin/Met), evaluated by using one-way analysis of variance (ANOVA) followed by Tukey’s multiple comparison test. Bar graph depicting mean ± standard error of mean (SEM) (*n* = 3); (**C**) Observed species depicting species diversity and evenness. Left: observed species showing the microbial diversity and richness for each treatment. The *x*-axis shows the number of valid sequences per sample and the *y*-axis shows the observed species (OTUs). Each curve in the graph represents a different group and is shown in a different color. Right: bar graph of the observed species at maximum OTUs, ### *p* < 0.001 compared to the control group (Ctrl), and * *p* < 0.05 and *** *p* < 0.001 compared to the antibiotic-treated groups (Clin/Met), evaluated by using one-way analysis of variance (ANOVA) followed by Tukey’s multiple comparison test. Data presented here is the mean ± standard error of mean (SEM) (*n* = 3).

In order to elucidate the structure and dynamics of the bacterial community in detail, we proceeded with the Illumina MiSeq (16S rRNA gene) platform [59]. For comparing the total diversity and species richness in different treatment groups, alpha diversity parameters were measured as reported in previous studies [60,61]. In our work, a total of 2,442,015 raw reads and 11,568 operational taxonomic units, (OTUs) ranging from 106 to 728 were obtained, out of which 2,254,503 clean tags were acquired from the 24 samples through Illumina miSequencing analysis. All the samples attained deep coverage (coverage = 1) (Supplementary Table S1). Rank abundance curve and rarefaction curves were constructed to identify and compare community richness and diversity in each treatment groups. All the curves at 97% sequence similarity as the OTU threshold showed notable differences in species diversity and richness of intestinal flora among the different treatment groups. The rank abundance curve thus generated directly demonstrated the species richness/abundance and the evenness in each treatment group (Figure 3A), where the richness and abundance of the species were reflected in the horizontal direction and by the width of the curve, respectively. The species richness from high to low between the groups was in the following order: control + DIP (Ctrl + DIP) > control (Ctrl) > metronidazole + DIP (Met + DIP) > clindamycin + DIP (Clin + DIP) > metronidazole + NR (Met + NR) > clindamycin + NR (Clin + NR) > clindamycin (Clin) > metronidazole (Met), furthermore highlighting the fact that antibiotic (Met/Clin) and the naturally restored groups (Clin + NR/Met + NR) had lower species richness. Another way to analyze species diversity and abundance is the rarefaction curves which reflects the number of sequences used in the analysis. As demonstrated in the Figure 3B,C, saturation plateau with different depths in each treatment was attained for all the curves suggesting the coverage of the majority of the microbial information in all the samples under investigation. Both curves (Shannon and Observed species) showed higher richness and diversity for control (Ctrl), control + DIP (Ctrl + DIP), and antibiotic + DIP-treated groups (Clin + DIP/Met + DIP) while species richness and diversity were reduced in antibiotic-treated (Met/Clin) and naturally restored groups (Clin + NR/Met + NR) similarly, as reflected through the rank abundance curve (Figure 3A). In addition to this, a box plot further supported the above-mentioned findings, indicating that the alpha-diversity indices were higher in the control (Ctrl) and DIP-treated groups (Clin + DIP/Met + DIP) in comparison with antibiotic (Clin/Met) and naturally restored groups (Clin + NR/Met + NR) (Supplementary Figure S2). Other alpha diversity indices evaluated in our study are presented in the additional information (Supplementary Table S2). Collectively, all these results suggest that the antibiotic-induced perturbation leads to a reduction in species richness and evenness; however, treatment with the polysaccharide (DIP) helps in modulating the bacterial community more efficiently in comparison with natural restoration by enhancing bacterial diversity and richness.

### 3.4. Beta Diversity Variations in Antibiotic and DIP Treated Mice

**Figure 4:**
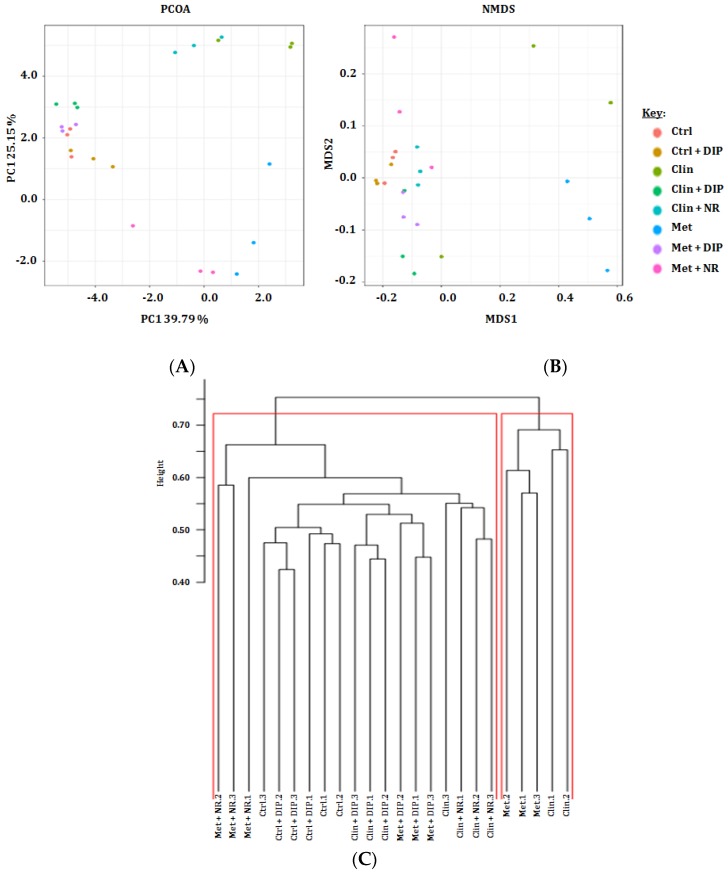
The representative graphical output of the beta diversity indices. (**A**) Principal coordinate analysis (PCoA) plot, (**B**) Non-metric multidimensional scaling plot (NMDS) with Bray–Curtis dissimilarity showing the distribution of the mice fecal samples based on the phylogenetic makeup of their microbiota. Each sample is represented by a dot in the graph, while different treatment groups are represented by different colors (*n* = 3). The distance between the points represents the level of difference; the closer the samples are, the higher the similarity between them. (**C**) Hierarchical cluster dendrogram representing similarities or dissimilarities between different treatment groups (*n* = 3 per group). Distance is shown on the scale bar.

Beta diversity analysis has been implicated to evaluate the differences between multiple groups [62]. To assess structural variability or similarities between different treatment groups in our investigation, a principal coordinate analysis (PCoA) and a non-metric multidimensional scaling (NMDS) plot, based on Bray-Curtis distances were generated. Based on these metrics, our results demonstrated a notable separation of antibiotic-treated (Clin/Met) and the naturally restored groups (Clin + NR/Met + NR) from the control (Ctrl) and DIP-treated groups (Clin + DIP/Met + DIP). DIP-treated groups depicted more similarities with the control group by clustering closely in principal coordinate analysis (PCoA) and non-metric multidimensional scaling (NMDS) plots (Figure 4A,B). A phylogenetic tree constructed to determine the variation of intestinal microflora among different treatment groups revealed greater similarity between the control (Ctrl) and DIP-treated groups (Clin + DIP/Met + DIP), while antibiotic (Clin/Met) and naturally restored groups (Clin + NR/Met + NR) were more distinct from the control (Ctrl) and DIP-treated groups (Clin + DIP/Met + DIP) (Figure 4C). In our investigation, beta diversity indices analysis highlighted the differences in the bacterial community structure where control (Ctrl) and DIP-treated groups (Clin + DIP/Met + DIP) have shown more similarity compared to the antibiotic (Clin/Met) and naturally restored groups (Clin + NR/Met + NR) that were clustered separately from the control and DIP-treated groups. These results suggested that the polysaccharide (DIP) has ameliorative effect on antibiotic-induced dysbiosis.

### 3.5. Alterations in Taxonomic Distribution Was Recovered by DIP Treatment in Antibiotic-Treated Mice 

**Figure 5:**
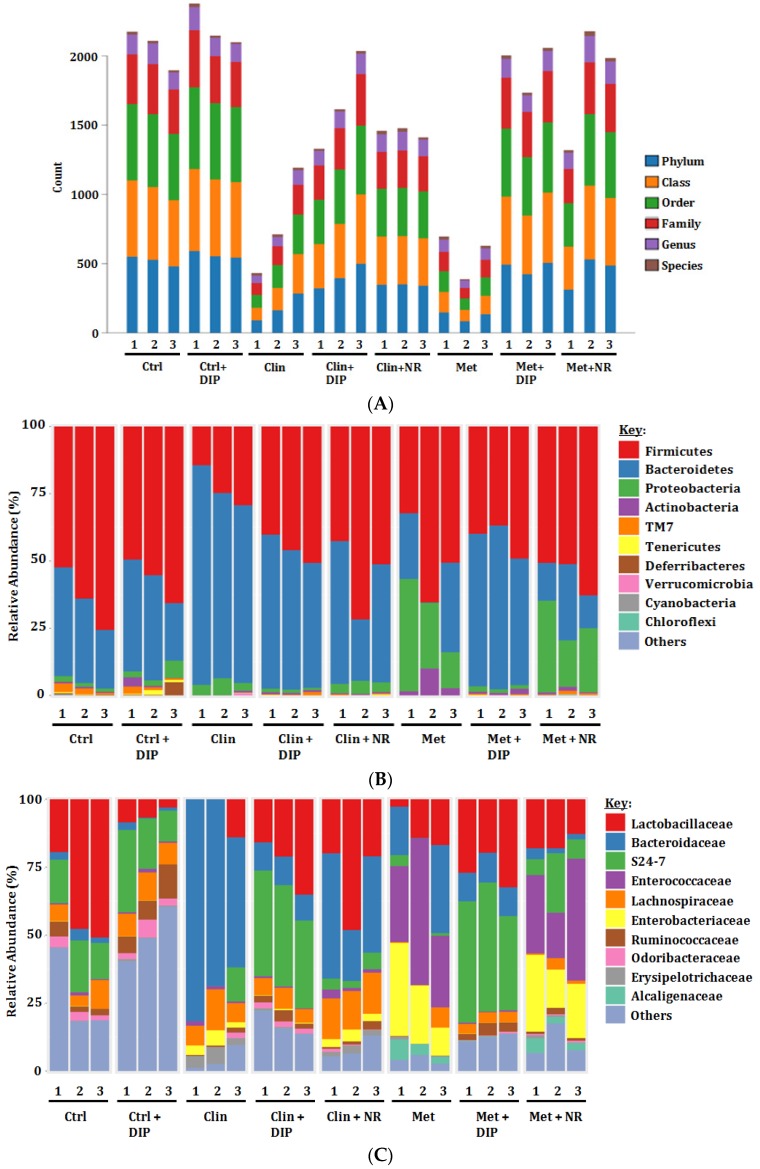
Taxonomic summary of fecal samples by Illumina miSequencing of 16S rRNA. (**A**) The taxonomic levels of phylum, class, order, family, genus, and species in individual samples (*n* = 3). The abscissa represents samples and the ordinate represents the number of OTUs. (**B**) Relative abundance (%) at the phylum level of each group under different treatment conditions (*n* = 3 per group). (**C**) Relative abundance (%) of the microbial community at the family level in different treatment groups (*n* = 3 per group).

In order to further investigate that DIP modulates the gut microbiota, we examined the relative abundance of the bacterial community by 16S rRNA gene sequencing after antibiotic-induced dysbiosis followed by DIP treatment at different taxonomic levels i.e., phylum, class, order, family, and genus. Data thus obtained showed that the antibiotic treatment has led to perturbation of the gut microbial community structure as indicated by the lower bacterial count in both antibiotic-treated groups (Clin/Met) as compared to the control group (Ctrl) (Figure 5A). At the phylum level, we found alterations in the abundance of three major phyla, Firmicutes, Bacteroidetes, and Proteobacteria in both the antibiotic-treated groups (Clin/Met) (Figure 5B). A drastic decrease in the proportion of Firmicutes (Clin 22.82% vs. Ctrl 64.04%) and an increase in the proportion of Bacteroidetes (Clin 72.16% vs. Ctrl 31.24%) was observed in the clindamycin-treated group (Clin). However, both phyla were lowered in the metronidazole-treated group (Met) i.e., Firmicutes (Met 50.87% vs. Ctrl 64.04%) and Bacteroidetes (Met 19.48% vs. Ctrl 31.24%) in comparison with the control group (Ctrl). It has been reported previously that the increased prevalence of Proteobacteria is associated with microbial imbalance (dysbiosis) [63] and serves as a potential diagnostic marker for several diseases e.g., metabolic [64] and inflammatory disorders [65]. In our study, the highly increased abundance of Proteobacteria was observed in the metronidazole-treated group as compared to the control group (Met 27.07% vs. Ctrl 1.53%). After 2 weeks of antibiotic cessation, surprisingly the ratio of Firmicutes/Bacteroidetes in the clindamycin naturally restored group (Clin + NR) almost returned to initial level but in metronidazole treatment group (Met + NR), the variation persisted. However, oral administration of DIP has efficiently restored the proportion of all the perturbed phyla, highlighting the clinical significance of DIP in the restoration of gut dysbiosis. The relative abundance of all the phyla in respective treatment is summarized in Supplementary Table S3. Furthermore, we proceeded with the evaluation of differences in the microbial community structure at a family level among different treatment groups. The variation in families across different groups upon respective treatment is presented in the Figure 5C. Antibiotic-treated groups (Clin/Met) varied from the control group with higher levels of Bacteroidaceae, Enterococcaceae (Met only), and Enterobacteriaceae, and decreased levels of Lactobacillaceae, Ruminococaceae, S24-7, and Odoribacteraceae. Supplementation with DIP reversed the trend by increasing Lactobacillaceae, Ruminococaceae, S24-7, and Odoribacteraceae and reducing Bacteroidaceae, Enterococcaceae (Met only), and Enterobacteriaceae in the antibiotic followed by DIP-treated groups (Clin + DIP/Met + DIP) compared to the naturally restored groups (Clin + NR/Met + NR) where bacterial flora remains perturbed. The bacterial abundance differences at family level are indicated in Supplementary Table S4. DIP administration reversed the dysbiosis and has shown the modulatory effect on gut microbiota community in antibiotic-treated groups (Clin + DIP/Met + DIP) as compared to the natural restored groups (Clin + NR/Met + NR) at phylum and family levels.

Next, we evaluated the relative abundance of bacterial communities at the genus level. Heatmap revealed the higher intensity of *Lactobacillus* in control (Ctrl) and DIP-treated groups (Clin + DIP/Met + DIP), while antibiotic-treated groups (Clin/Met) showed slightly lower intensity (Supplementary Figure S3). In harmony with the heatmap, the bubble plot also depicted the similar results, i.e., *Lactobacillus* was increased in DIP-treated groups (Clin + DIP/Met + DIP) as compared to antibiotic-treated (Clin/Met) and naturally restored groups (Clin + NR/Met + NR) (Supplementary Figure S4). Furthermore, an increment of *Bacteroides* was observed in clindamycin-treatment (Clin) and *Enterococcus* was drastically increased in metronidazole-treatment (Met). However, DIP treatment reversed the trend compared to naturally restored groups (Clin + NR/Met + NR). This outcome of our study highlighted the protective effect of DIP as several animal studies have suggested that some of the *Lactobacillus* strains including *Lactobacillus rhamnosus GG (LGG)* possess probiotic properties that have a modulatory effect on immune system, decrease inflammatory conditions, and provide protection against certain intestinal infections [66,67].

### 3.6. Effect of DIP on Histological Perturbation in the Colon Tissue of Antibiotic Treated Mice

**Figure 6:**
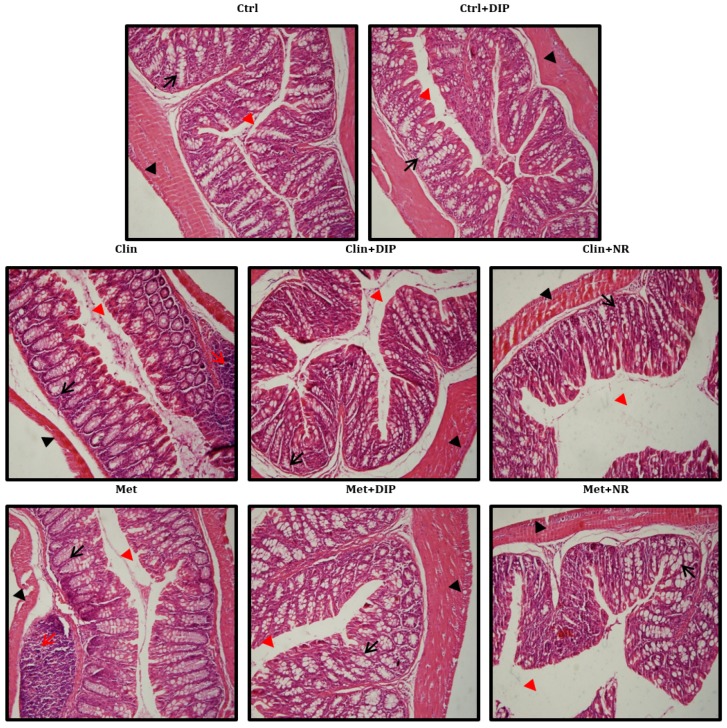
Effect of DIP on colonic histological alterations in antibiotic-induced dysbiosis mice. Photomicrographs of hematoxylin and eosin (H&E)-stained distal colon sections. Shown are inflammatory cells (indicated with red arrows), the mucosal space (red arrow head), goblet and epithelial cells (black arrows), and the epithelium surface (black arrow head). Original magnification ×20, Scale bar: 100 µm.

The histological evaluation of colonic tissue of mice in different treatment groups was done by H&E staining (Figure 6) which revealed a normal histology of the mice in the control group (Ctrl) having well-shaped, compact, and elongated villi and crypt lined with dark-stained nuclei in lamina propria. In contrast, antibiotic-treated mice (Clin/Met) exhibited histological alterations with blunted, short, irregular villi and a deformed epithelial barrier. Inflammatory cell infiltrates were also observed with disintegrated epithelial cells and loss of crypt and goblet cells in both antibiotic groups (Clin/Met), with a pronounced increase in the space between mucosa and submucosa indicating that the antibiotic exposure induced colonic inflammation. However, DIP-treated mice (Clin + DIP/Met + DIP) reduced the histological loss of colon wall structure and improved the extent and the severity of the macroscopic and histological signs of colon inflammation in comparison with antibiotic-treated (Clin/Met) and naturally restored groups (Clin + NR/Met + NR).

### 3.7. DIP Decreases Pro-Inflammatory Cytokine Levels in Antibiotic-Induced Inflammation

**Figure 7:**
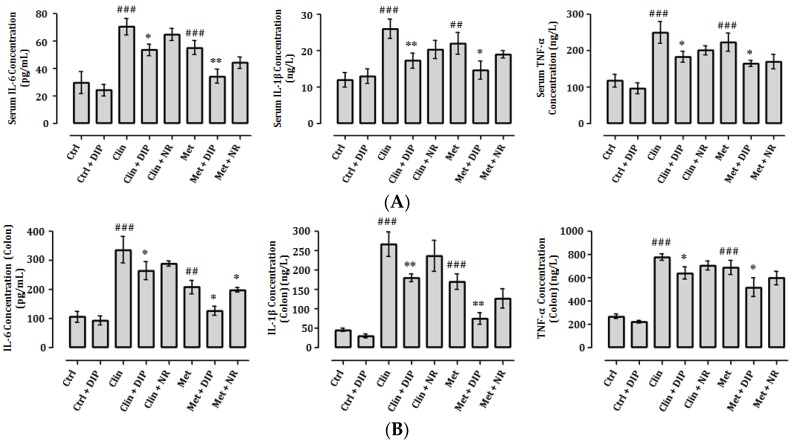
Pro-inflammatory cytokine (IL-6, IL-1β, and TNF-α) measurement with ELISA in serum and colon. The levels of Pro-inflammatory cytokines in (**A**) serum and (**B**) colon. The data are presented as the means ± standard error of mean SEM (*n* = 6). Statistical significance was assessed by one-way analysis of variance (ANOVA) followed by Tukey’s multiple comparison test and is represented as follows: ## *p* < 0.01 and ### *p* < 0.001 compared to the control; * *p* < 0.05, ** *p* < 0.01 compared to the respective antibiotic (Clin/Met)-treated group). IL: interleukin; TNF: tumor necrosis factor.

Induction of inflammation and the production of pro-inflammatory cytokines including tumor necrosis factor-alpha (TNF-α), interleukin-1-beta (IL-1β), and interleukin-6 (IL-6) as a result of antibiotic-induced intestinal dysbiosis has been reported previously [68]. We therefore measured the levels of these cytokines in the serum and colon of different treatment groups. Consistent with the previous findings, in our study pro-inflammatory cytokines were higher in antibiotic-treated groups (Clin/Met). DIP treatment reduced the levels of inflammatory cytokines in antibiotic-treated mice (Clin + DIP/Met + DIP) as compared to the natural restored groups (Clin + NR/Met + NR) (Figure 7A,B). These results indicate that DIPs could reduce the antibiotic-induced inflammation.

### 3.8. DIP Reduces Endotoxemia and Enhance Intestinal Tight Junctions in Antibiotic-Treated Mice

**Figure 8:**
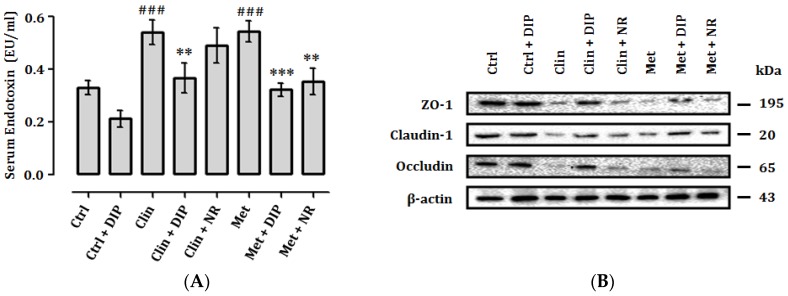
DIP treatment reduced serum endotoxin (lipopolysaccharide, LPS) levels and increased intestinal tight junctions in antibiotic-treated mice. (**A**) Effect of DIP on antibiotic-induced endotoxemia. Serum endotoxin (EU mL^−1^) was measured using a Limulus amoebocyte lysate assay kit. LPS levels were higher in antibiotic-treated groups (Clin/Met) as compared to the Ctrl group. The data are presented as the means ± standard error of mean (SEM) (*n* = 6). Statistical significance was assessed by one-way analysis of variance (ANOVA) followed by Tukey’s multiple comparison test and is represented as follows: ### *p* < 0.001 compared to control, ** *p* < 0.01, *** *p* < 0.001 compared to the respective antibiotic group (Clin/Met)). (**B**) Effect of DIP on tight-junction protein expression in the colon. Expression of claudin, occludin, and zonula occludens-1 (ZO-1) in different treatment groups using β-actin as an internal control. Western blots are representative of three independent experiments.

Gut permeability is indicated by the increased levels of endotoxins such as LPS in the serum and expression of tight-junction proteins. Previously it has been reported that antibiotic administration increases serum endotoxin (LPS) levels and reduces the expression of tight junction proteins that consequently impairs the gut permeability [68]. We examined the effect of broad-spectrum antibiotics (clindamycin and metronidazole) on LPS levels in the serum and expression of tight-junction proteins in the colon (Figure 8A,B). A higher level of LPS was observed in antibiotic-treated groups (Clin/Met) compared to the control (Ctrl). DIP treatment reduced the LPS levels in antibiotic-treated groups (Clin + DIP/Met + DIP) on the contrary to natural restored groups (Clin + NR/Met + NR) where LPS levels were high in comparison with DIP-treated groups. On the other hand, there was a lower expression of tight junction proteins in antibiotic-treated groups (Clin/Met) compared to the control (Ctrl) group. Oral administration of DIP modulated the expression of tight junction proteins in antibiotic-treated groups (Clin + DIP/Met + DIP) compared to the naturally restored groups (Clin + NR/Met + NR). These findings suggested that DIP may improve intestinal barrier integrity in an antibiotic-induced dysbiosis mouse model.

### 3.9. Antibiotic-Driven Dysbiosis Diminished Mucus Layer and Decreased Mucin-2(MUC2) Protein Expression in the Colon That is Modulated by DIP Treatment

**Figure 9:**
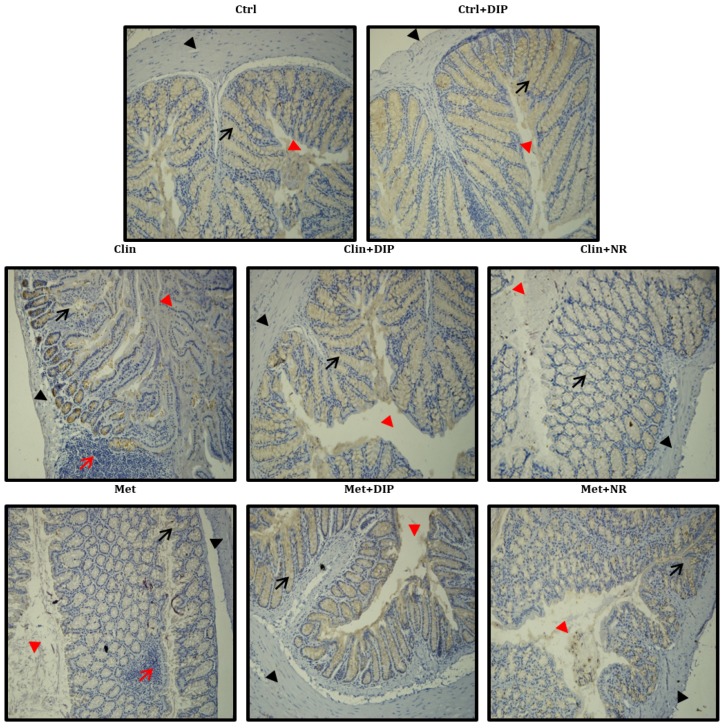
Representative immunohistochemistry staining for Mucin-2 in the colon of eight different treatment groups. Shown are mucin expression (indicated with black arrows), inflammatory cells (red arrows), the mucosal space (red arrow head), and the epithelium surface (black arrow head). Original magnification ×20, Scale bar: 100 µm.

Mucin-2 is the prominent component of gut barrier epithelium that is secreted from goblet cells [69]. It has been reported previously that antibiotic exposure results in the intestinal microbial community imbalance which may cause a reduction in goblet cells, decreases the MUC2 protein expression, and thinning of the inner mucus layer [31,70]. We performed immunohistochemistry (IHC) to visualize the gut integrity and MUC2 protein expression pattern after antibiotic exposure for two weeks followed by DIP treatment (Figure 9). In our study antibiotic treatment (Clin/Met) caused a reduction in the mucus layer thickness and decreased MUC2 protein expression. The structure of columnar epithelial cells were damaged, and the influx of inflammatory cells was evidenced after antibiotic exposure in both antibiotic-treated (Clin/Met) groups. In addition, the average number of MUC2 positive cells in antibiotic-exposed groups (Clin/Met) were apparently reduced. Compared to the metronidazole-treated group (Met), the clindamycin-treated group (Clin) depicted greater severity with almost complete destruction of columnar epithelial and goblet cells. However, DIP treatment dramatically increased the mucus layer thickness, improved epithelial cells, and enhanced MUC2 protein expression in antibiotic-treated groups (Clin + DIP/Met + DIP) compared to the naturally restored (Clin + NR/Met + NR) groups. The above findings led us to infer that DIPs may attenuate antibiotic-induced inflammation and augment host defense via enhanced MUC2 protein expression and increased mucus layer production.

## 4. Discussion

Mushrooms are widely consumed due to its health-promoting properties. Several lines of evidence depicted the clinical significance of mushroom polysaccharides [71,72,73,74] including the polysaccharides isolated from the mushroom *Dictyophora indusiata* [50,51,52,53]; however, the impact of polysaccharides from mushrooms in general and from *D. indusiata* in particular on gut microbial ecology has been rarely investigated. In this study, our data revealed that the DIP supplementation restored the gut microbiota community structure, enhanced microbial diversity, reduced the intestinal inflammation, and ameliorated the gut barrier integrity caused by antibiotic administration.

We isolated the crude polysaccharide (DIP) from the fruiting body of mushroom *D. indusiata* with a total sugar content of 96.66%. The monosaccharide composition analysis showed the bioactive moieties were glucose, mannose, and galactose (59.84%, 23.55%, and 12.95%, respectively). Furthermore, dysbiosis was induced with two broad-spectrum antibiotics (clindamycin (0.2 mg/0.2 mL)/metronidazole (3.5 mg/0.2 mL)) and the protective effect of the DIP was investigated using BALB/c mice as a model system. Initially, the bacterial diversity perturbation in the gut after antibiotic exposure and its restoration upon DIP (0.2 mg/0.2 mL) treatment was determined by PCR-DGGE followed by 16S rRNA Illumina MiSeq technique for deep analysis.

The reduction in gut microbiota diversity and richness is associated with the etiology of inflammatory as well as neuronal disorders such as Crohn’s disease, irritable bowel syndrome, colorectal cancer and autism [4,75,76,77]. We analyzed the alpha and beta diversity indices to investigate microbial diversity, richness, and determined differences between different treatment groups. Alpha diversity has shown reduced microbial diversity and richness upon antibiotic (Met/Clin) exposure that was reversed and enhanced after DIP administration (Figure 3). Dissimilarities among different treatments were elucidated via beta diversity indices which exhibited that the antibiotic-treated groups (Met/Clin) deviated from the control group, whereas the DIP-treated (Clin + DIP/Met + DIP) and the control group were clustered, closely reflecting more similarities among each other (Figure 4). These results suggested that DIP may exert a positive effect on gut microbiota ecology and enhance bacterial diversity and richness.

In order to investigate bacterial abundance at the different taxonomic levels, we evaluated the composition of flora at the phylum, family, and genus level. At the phylum level, in the control group the proportion Firmicutes was greater than that of Bacteroidetes, whereas the antibiotic treatment (Clin/Met) remarkably perturbed the intestinal flora structure. However; the trend in both the antibiotics was different. In clindamycin treatment, the proportion of Firmicutes/Bacteroidetes was decreased as compared to the control group. However, DIP treatment (Clin + DIP) reversed these alterations at more or less same pace as the naturally restored group and depicted similar Firmicutes/Bacteroidetes proportions to the control group. On contrary, metronidazole treatment showed reduced Firmicutes and Bacteroidetes levels and increased abundance of Proteobacteria as compared to the control group. Surprisingly, DIP treatment (Met + DIP) enhanced the Bacteroidetes level and reduced the level of Firmicutes and Proteobacteria. Meanwhile, the naturally restored group failed to decrease the higher levels of Proteobacteria that has been implicated previously with the microbial imbalance and progression of diseases [63]. Moreover, an increased abundance of *Bacteroides* in clindamycin treatment (Clin) and higher levels of *Enterococcus* were observed in metronidazole treatment (Met) (Supplementary Figure S4). Elevated levels of *Bacteroides* have been previously associated with increased enterotoxin secretion [78] and *Enterococcus* species such as *Enterococcus faecalis* have been reported to induce inflammatory diseases like IBD [79,80]. The expansion of *Enterococcus* has been also associated with hepatic inflammation and development of liver disease [81] and is most commonly capable of causing urinary tract infections and a variety of community-acquired infections [82] Nonetheless, the notable improvement was observed after DIP administration in antibiotic treated groups (Clin + DIP/Met + DIP) in comparison with the natural restoration (Clin + NR/Met + NR) suggesting the ameliorative properties of DIP in decreasing pathogenic flora. On the other hand, health modulating flora such as, *Lactobacillus* was enriched in control and DIP-treated groups (Clin + DIP/Met + DIP) (Supplementary Figure S4), that has been shown to possess immunoregulatory [83] and anti-inflammatory properties in colitis onset [84,85,86,87]. The structure and abundance of gut microbiota could be restored by dietary manipulation such as mushroom polysaccharide (DIP) which not only helps in the restoration of gut microbiota but also encourages the growth of beneficial flora like Lactobacillaceae (lactic acid-producing bacteria), and Ruminococcaceae (butyrate-producing bacteria) flora (Figure 5C, Supplementary Figure S4 and Supplementary Table S4) and also decrease the abundance of harmful bacteria such as *Enterococcus, Bacteroides*, and Proteobacteria (Supplementary Figure S4 and Figure 5B). Collectively, these findings at various taxonomic levels suggest that oral administration of DIP could modulate gut microbiota ecology by reverting dysbiosis.

In order to investigate the association of broad-spectrum antibiotic driven gut dysbiosis and DIP-mediated restoration on the host physiology and its ultimate exposure to the development and prevention of disease(s), respectively, we elucidated the changes in colon histology, intestinal permeability, inflammatory responses, and expression of tight-junction proteins after the exposure to antibiotics and DIP treatments. Our data illustrated that antibiotic-induced dysbiosis has profound effects on the gut morphology and intestinal permeability. The histological analysis revealed mucosal erosion, inflammatory cell infiltration, reduced villus height, loss of crypts, and lower goblet cells in antibiotic-treated groups, whereas polysaccharide (DIP) administration improved gut morphology (Figure 6). Furthermore, antibiotic treatment resulted in elevated LPS levels suggesting increased endotoxemia that was lowered after DIP treatment (Figure 8A). A correlation between the elevated levels of *Bacteroides* and enhanced enterotoxin production has been reported previously that increases the intestinal permeability and bacterial penetration [78]. Consistent with this study, we also observed the higher abundance of *Bacteroides* in antibiotic-treated group (Clin) that was reverted back and even decreased after DIP treatment (Supplementary Figure S4). This increase and decrease in the abundance of *Bacteroides* in the antibiotic- and DIP-treated groups, respectively, are positively correlated with the increase and decrease level of endotoxins.

It has been reported previously that the intestinal injury and inflammatory responses can enhance the expression of pro-inflammatory cytokines in the intestine [88,89] where the excessive/enhanced production of pro-inflammatory cytokines has been negatively correlated with intestinal mucosal integrity and physiology [90,91]. Antibiotic-induced dysbiosis in mice model promotes intestinal inflammation and thus produce higher levels of pro-inflammatory cytokines including tumor TNF-α, IL-1β, MCP-1, and IFN-γ [68,92,93,94]. In parallel with these studies, we also observed there was an increased abundance of the Bacteroidetes (Clin) and Proteobacteria (Met) at the phylum level that are positively associated with increased production of pro-inflammatory cytokines. However, DIP administration significantly reduced the production of pro-inflammatory cytokines in antibiotic-treated groups (Clin + DIP/Met + DIP) (Figure 7A,B) and ameliorated the inflammatory response by modulating gut microbiota ecology. From these findings, we can infer that DIPs could effectively reduce antibiotic-induced inflammation and enhance immunity.

Furthermore, we investigated the tight junction protein expression in the gut. The intercellular tight-junction proteins known as TJs such as zonula occludens-1 (ZO-1), claudin-1, and occludin play a vital role in maintaining the intestinal barrier integrity [95,96]. These proteins maintain the gut epithelial integrity and regulate tight junction structure [97,98,99] while reduced expression of TJs enhances the gut epithelial permeability [100]. In our study, antibiotic-induced dysbiosis led to decreased tight junction protein expression, in particular occludin, which has apparently shown lower expression as compared to ZO-1 and claudin-1. The protective effects of dietary supplements such as mushroom polysaccharides could reduce the inflammatory responses and enhance the immunity [71,101]. In concordance with these studies, our findings also revealed that the polysaccharide treatment (DIP) enhanced the TJs expression level in DIP-treated groups (Clin + DIP/Met + DIP); nonetheless, the expression level of occludin was slightly lower relative to ZO-1 and claudin-1 (Figure 8B). Hypothetically, this could be possibly because of the dynamic structure and localization of tight junction proteins that undergo continuous remodeling and some of the proteins get rapidly diffused within the tight junction [102]; or higher expression of inflammatory cytokine could also reduce the expression of TJ’s by triggering inflammatory processes [103]. To validate further that dietary modulation such as use of polysaccharides can enhance gut barrier integrity, we examined one of the main components of gut barrier protein mucin-2 (MUC2) expression via immunohistochemistry (IHC) (Figure 9). We found that broad-spectrum antibiotics disrupted the intestinal mucosal layer and drastically decreased MUC2 expression. In contrast, DIP administration improved the gut epithelial barrier by increasing mucus-producing goblet cells and MUC2 expression and ameliorated the structure of colon tissue. The above findings led us to infer that DIP Polysaccharide may attenuate antibiotic-induced inflammation, reduced endotoxemia, and augment host defense via modulation of gut microbiota, elevated expression of tight-junction proteins, and enhanced MUC2 protein expression.

## 5. Conclusions

In conclusion, based on the Illumina MiSeq platform and biochemical analysis, our data suggested that the administration of broad-spectrum antibiotics (clindamycin and metronidazole) in the healthy mice resulted in the gut dysbiosis that is associated with the intestinal lesions, decreased expression of tight-junction associated proteins, and increased levels of endotoxin and pro-inflammatory cytokines. Treatment with the crude polysaccharide (DIP) from the mushroom *D. indusiata* has shown a protective effect by reverting the antibiotic-induced dysbiosis and modulated the gut microbiota ecology by reducing pathogenic bacteria such as *Enterococcus*, *Bacteroides* and Proteobacteria and increasing beneficial flora such as, Lactobacillaceae (lactic acid-producing bacteria) and Ruminococaceae (butyrate-producing bacteria) at various taxonomic levels. Taken together, these results revealed that DIP could be used as a prebiotic source that has a modulatory effect on gut microbiota ecology and improved the intestinal integrity by increasing and decreasing the expression of tight-junction proteins and endotoxin/pro-inflammatory cytokine levels, respectively. However, our study emphasizes the need for a detailed investigation into the purified polysaccharide fraction and species-level study in order to fully understand its role in the restoration of gut microbiota/barrier function and near future clinical implication as dietary therapeutics.

## Figures and Tables

**Table 1:** Chemical and monosaccharide composition of *D. indusiata* crude polysaccharide (DIP).

Chemical Composition		
Yield of crude polysaccharide (*w*/*w*)%	13.2	
^1^ Total sugar content%	96.66	
^2^ Total protein content%	2.38	
^3^ Monosaccharide composition		
Components	mg/Kg	%
Mannose	35160.22	23.55
Ribose	694.41	0.46
Rhamnose	65.2	0.043
Glucuronic acid	1515.23	1.014
Galacuronic acid	^4^ ND	ND
Glucose	89,350.23	59.84
Galactose	19,350.23	12.95
Xylose	544.02	0.36
Arabia sugar	257.26	0.17
Fucose	2372.73	1.58

^1^ Phenol-H_2_SO_4_ method; ^2^ Bicinchoninic acid method; ^3^ High performance liquid chromatography (HPLC); ^4^ ND, not detected.

## Supplementary Materials

The following are available online at http://www.mdpi.com/2072-6643/10/8/1003/s1, Figure S1: PCR-DGGE profiles of fecal samples using 16S rRNA gene, Figure S2: Box plot of richness estimators of the gut microbiota of mice in each treatment to determine differences in bacterial community diversity and richness, Figure S3: Hierarchical clustering of the gut microbiome, Figure S4: Bubble plots showing the relative abundances of the top ten abundant genera in mice fecal sample, Table S1: Summary of OTU sequencing data attained in this study, Table S2: Summary of alpha diversity indices estimation via 16S rRNA gene sequencing, Table S3: Percentage of bacterial phyla in different treatment groups, Table S4: Percentage of bacterial family in different treatment groups.
