# Supplementary material for: A Polysaccharide Isolated from Dictyophora indusiata Promotes Recovery from Antibiotic-Driven Intestinal Dysbiosis and Improves Gut Epithelial Barrier Function in a Mouse Model
Source: Nutrients. 2018 Jul 31;10(8):1003. doi: 10.3390/nu10081003 (PMC6115818; doi:10.3390/nu10081003)
Supplement: Supplementary file 1 [file nutrients-10-01003-s001.pdf]

A Polysaccharide Isolated from *Dictyophora indusiata* Promotes Recovery from Antibiotic-Driven Intestinal Dysbiosis and Improves Gut Epithelial Barrier Function in Mice Model

Sadia Kanwal, Thomson Patrick Joseph, Lawrence Owusu, Ren Xiaomeng, Li Meiqi and Xin Yi

Supplementary Information

Supplementary Figures
Figure S1

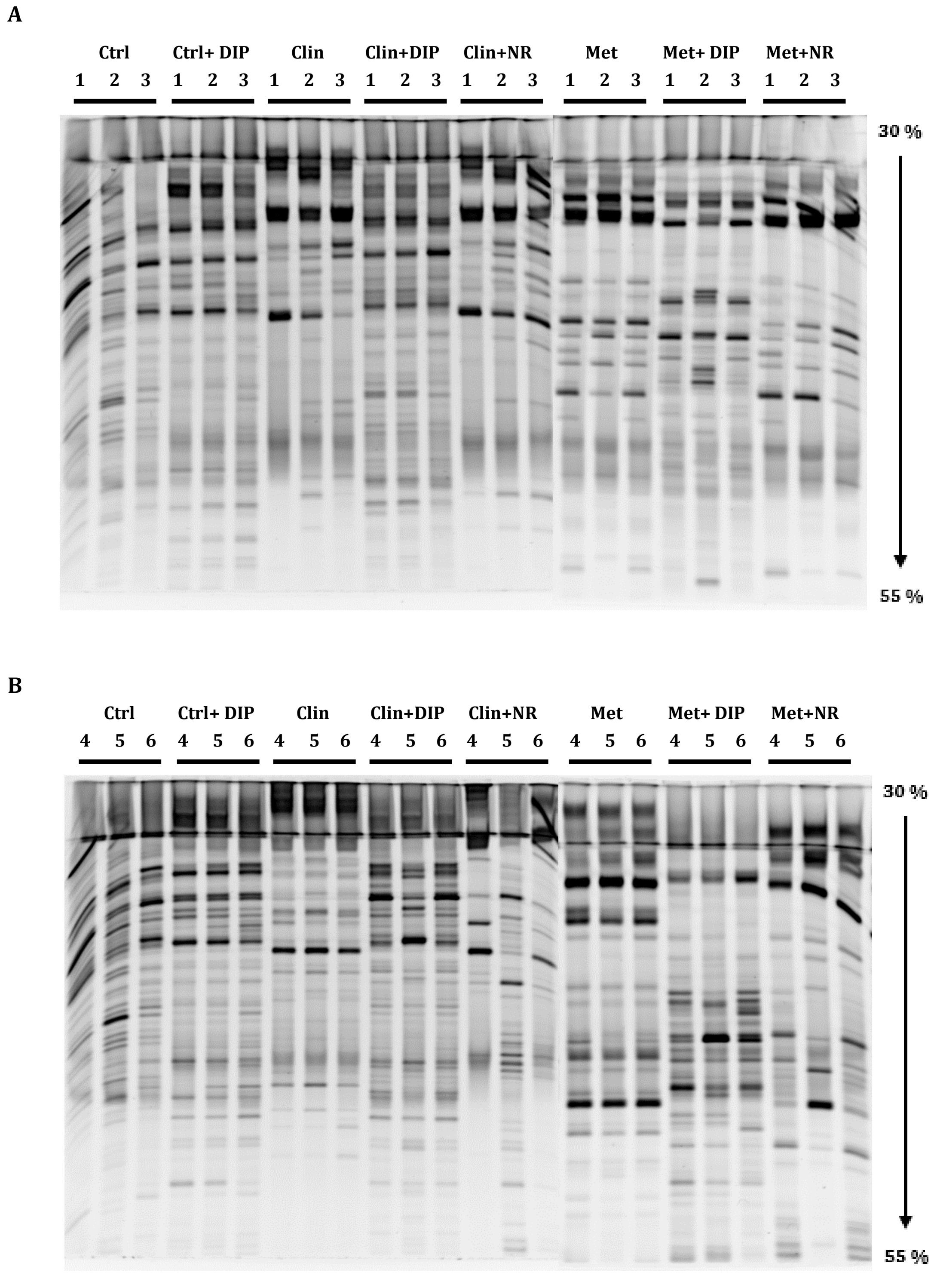

Figure S1: **PCR-DGGE profiles of fecal samples using 16S rRNA gene.** DGGE analysis of microbial diversity across different treatment groups. Bacterial fingerprint profile is based on 30 to 55% DGGE gel gradient, representing each treatment(n=6). **(A)** 1-3, **(B)** 4-6.

Figure S2

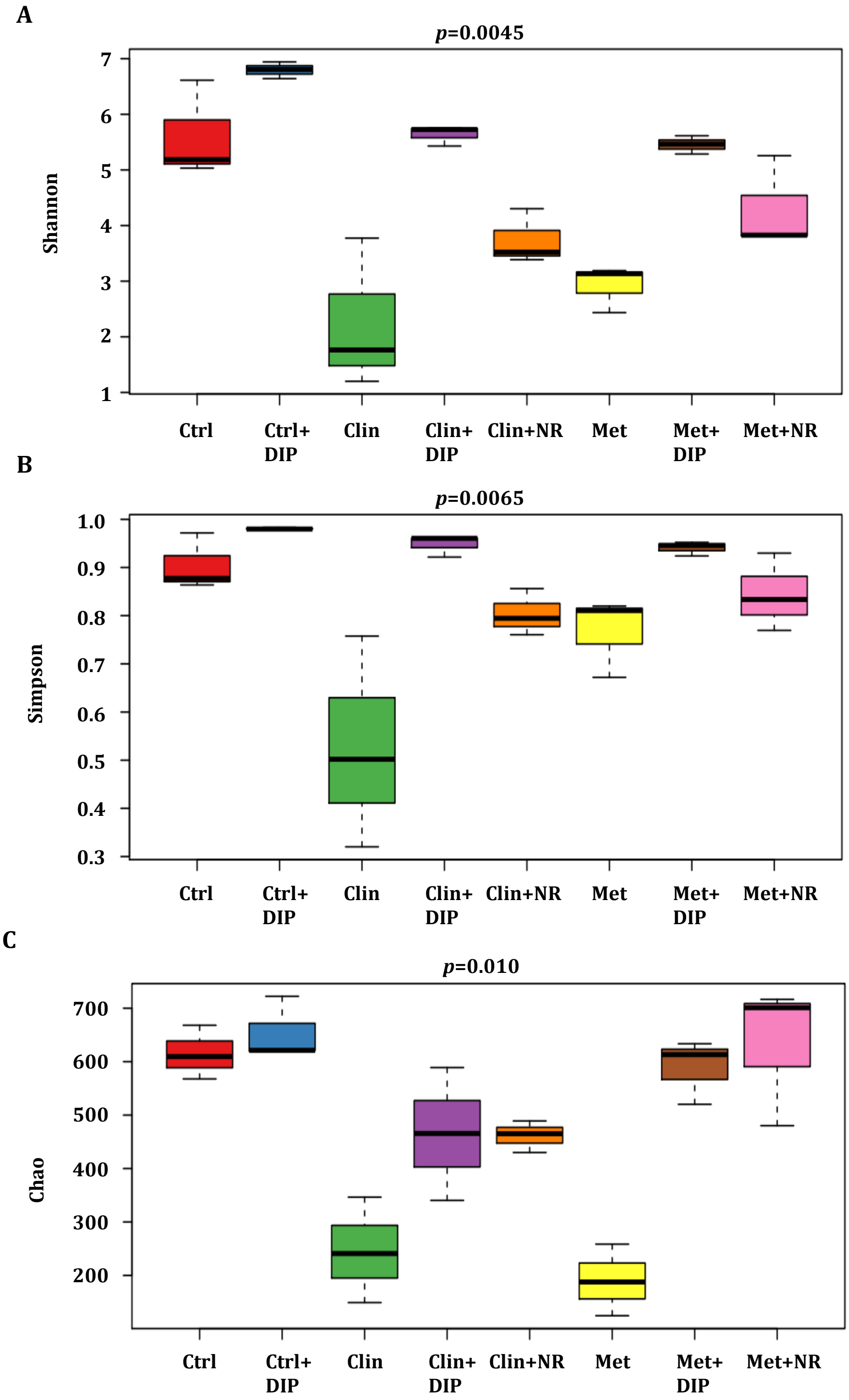

Figure S2: **Box plot of richness estimators of the gut microbiota of mice in each treatment to determine differences in bacterial community diversity and richness.** Boxes represent the interquartile range (IQR) between the first and third quartiles (25th and 75th percentiles, respectively), and the horizontal line inside the box defines the median. Whiskers represent the lowest and highest values within 1.5 times the IQR from the first and third quartiles, respectively. *$p < 0.05$ (Student's t-test). Shannon index (p=0.0045), Simpson index (p=0.0065) and Chao1 (p=0.010)

Figure S3

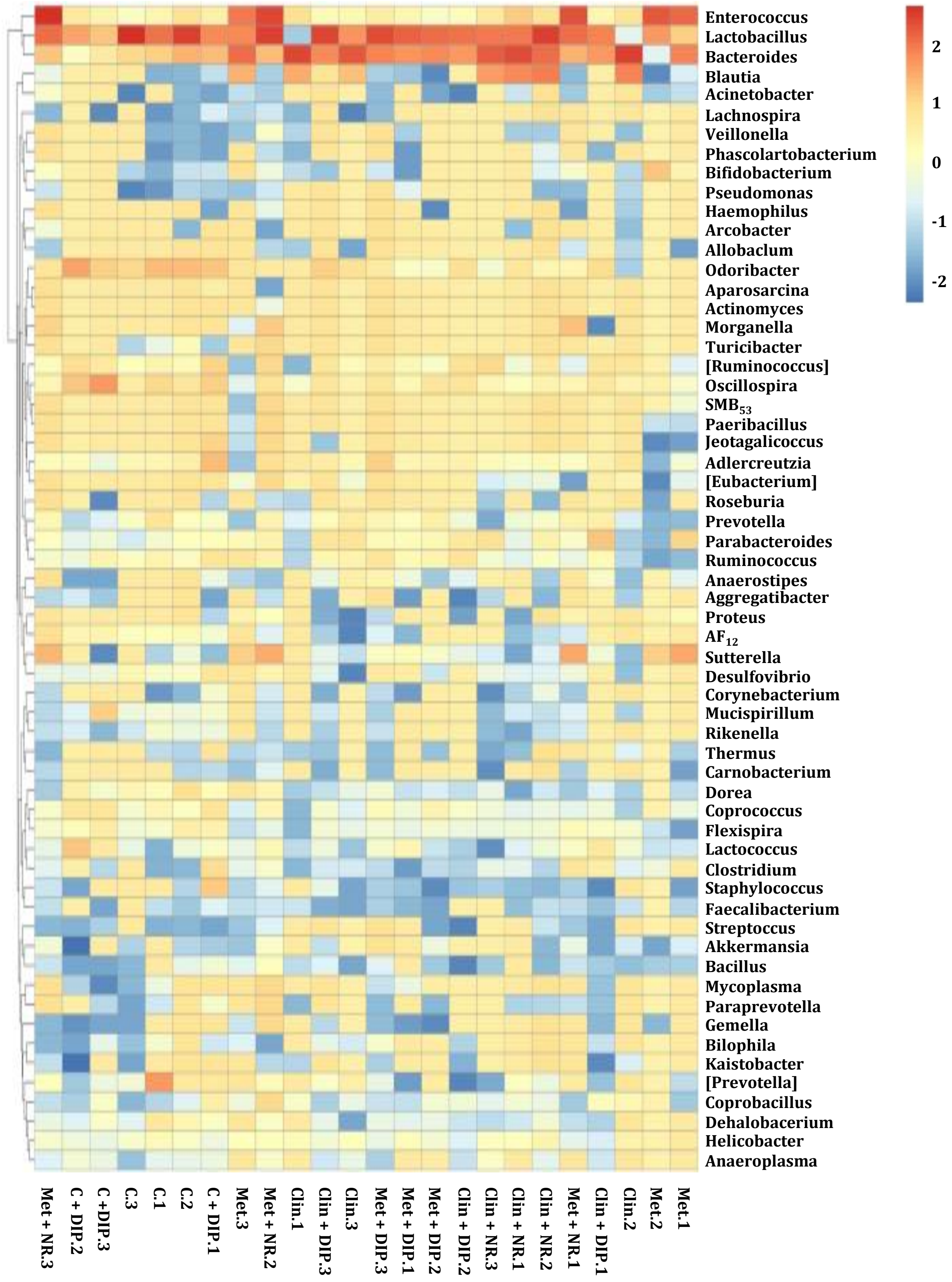
Enterococcus
Lactobacillus
Bacteroides
Blautia
Acinetobacter
Lachnospira
Veillonella
Phascolartobacterium
Bifidobacterium
Pseudomonas
Haemophilus
Arcobacter
Allobaclum
Odoribacter
Aparosarcina
Actinomyces
Morganella
Turicibacter
[Ruminococcus]
Oscillospira
SMB53
Paeribacillus
Jeotagalicoccus
Adlercreutzia
[Eubacterium]
Roseburia
Prevotella
Parabacteroides
Ruminococcus
Anaerostipes
Aggregatibacter
Proteus
AF12
Sutterella
Desulfovibrio
Corynebacterium
Mucispirillum
Rikenella
Thermus
Carnobacterium
Dorea
Coprococcus
Flexispira
Lactococcus
Clostridium
Staphylococcus
Faecalibacterium
Streptoccus
Akkermansia
Bacillus
Mycoplasma
Paraprevotella
Gemella
Bilophila
Kaistobacter
[Prevotella]
Coprobacillus
Dehalobacerium
Helicobacter
Anaeroplasma
2
1
0
-1
-2
Met + NR.3
C + DIP.2
C +DIP.3
C.3
C.1
C.2
C + DIP.1
Met.3
Met + NR.2
Clin.1
Clin + DIP.3
Clin.3
Met + DIP.3
Met + DIP.1
Met + DIP.2
Clin + DIP.2
Clin + NR.3
Clin + NR.1
Clin + NR.2
Met + NR.1
Clin + DIP.1
Clin.2
Met.2
Met.1

Figure S3: Hierarchical clustering of gut microbiome. Heat map analysis of the highly characterized bacterial taxa at the genus level. Heat map indicating genus-level changes among Ctrl, Ctrl+DIP, Clin, Clin+DIP, Clin+NR, Met, Met+DIP, and Met+NR groups. Genera and subjects are listed to the right and x-axis respectively. The relative abundance of each genus is indicated by a gradient of color from blue (low abundance) to red (high abundance). The data represented twenty-four samples from respective groups.

Figure S4

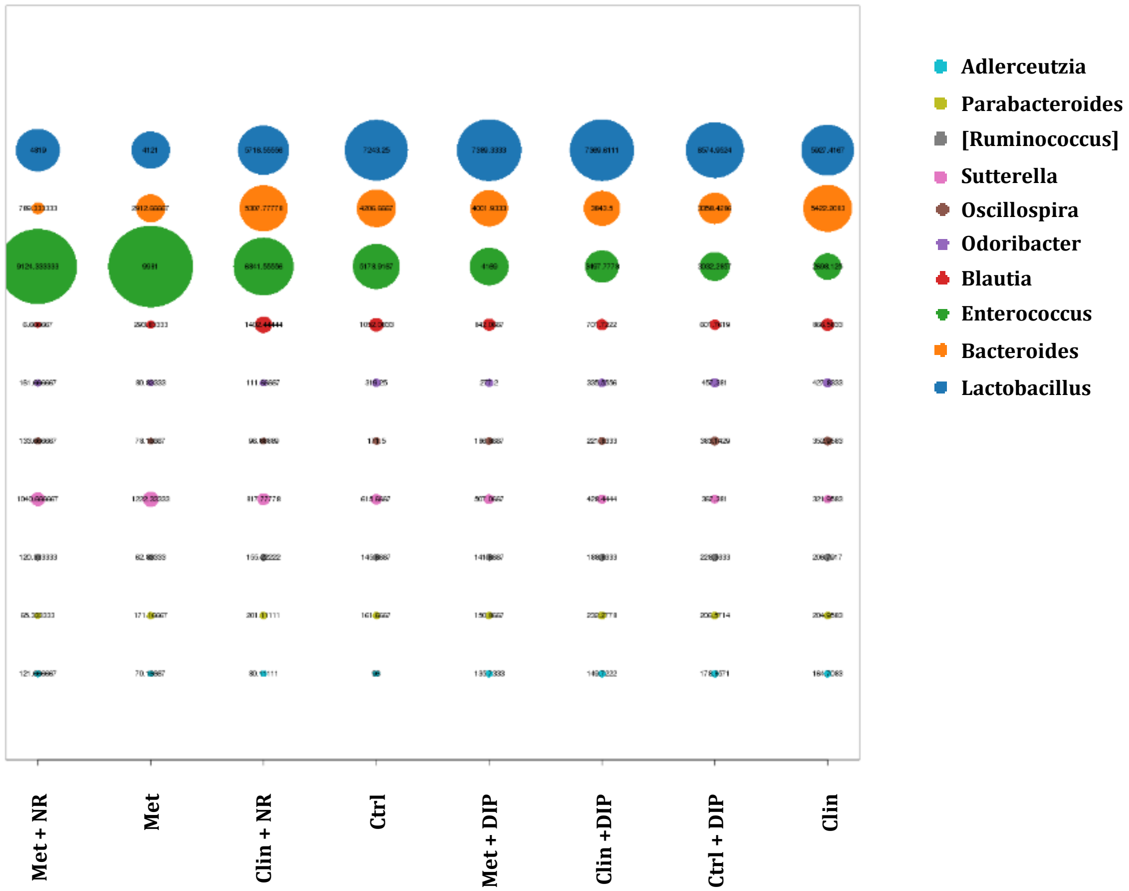

Figure S4: Bubble plots showing the relative abundances of the top ten abundant genera in mice fecal sample. Lactobacillus, Bacteroides and Enterococcus genera were abundant genera in different treatment groups.

Supplementary Tables

Table S1: Summary of OTUs sequencing data attained in this study.

Groups	Sample Size	OTUs Number	OTUs Sequence	Coverage
**Ctrl.1**	101171	646	92106	1
**Ctrl.2**	104909	659	96987	1
**Ctrl.3**	101593	582	93053	1
**Ctrl + DIP.1**	104564	728	96383	1
**Ctrl + DIP.2**	102257	649	91994	1
**Ctrl + DIP.3**	99975	656	91355	1
**Clin.1**	102532	149	98523	1
**Clin.2**	98864	267	94492	1
**Clin.3**	100498	372	92840	1
**Clin + DIP.1**	75716	335	65457	1
**Clin + DIP.2**	109280	494	99529	1
**Clin + DIP.3**	104635	588	94103	1
**Clin + NR.1**	101506	500	94330	1
**Clin + NR.2**	113138	476	108404	1
**Clin + NR.3**	98767	444	93769	1
**Met.1**	101915	210	95526	1
**Met.2**	98281	106	89672	1
**Met.3**	101182	166	92035	1
**Met + DIP.1**	98091	609	88991	1
**Met + DIP.2**	103034	540	94985	1
**Met + DIP.3**	105386	651	97300	1
**Met + NR.1**	108709	414	99314	1
**Met + NR.2**	100231	688	95333	1
**Met + NR.3**	105781	639	98022	1

Table S2: Summary of alpha diversity indices estimation via 16S rRNA gene sequencing.

Groups	Shannon	Simpson	Chao1	Ace	Goods Coverage
**Ctrl.1**	6.6122	0.9718	609.4783	615.8543	0.9971
**Ctrl.2**	5.0317	0.8637	668.0000	667.0465	0.9954
**Ctrl.3**	2.1839	0.8772	567.6154	576.2996	0.9965
**Ctrl + DIP.1**	6.9417	0.9832	722.1667	699.2784	0.9958
**Ctrl + DIP.2**	6.6401	0.9792	620.4000	620.5705	0.9969
**Ctrl + DIP.3**	6.8051	0.9798	621.1231	627.3995	0.9967
**Clin.1**	1.2000	0.3202	149.2353	162.9668	0.9985
**Clin.2**	1.7624	0.5020	241.0000	290.0961	0.9974
**Clin.3**	3.7733	0.7576	346.4419	350.0430	0.9976
**Clin + DIP.1**	5.7558	0.9610	340.3704	337.9169	0.9989
**Clin + DIP.2**	5.7258	0.9606	465.6250	464.8409	0.9972
**Clin + DIP.3**	5.4292	0.9216	588.8800	601.3334	0.9962
**Clin + NR.1**	3.3866	0.7605	465.0984	497.5626	0.9960
**Clin + NR.2**	3.5209	0.7945	489.0000	457.3468	0.9965
**Clin + NR.3**	4.3022	0.8562	430.0714	428.7069	0.9971
**Met.1**	3.1347	0.8199	258.6250	240.0515	0.9980
**Met.2**	2.4335	0.6719	124.6250	110.5388	0.9991
**Met.3**	3.1888	0.8106	187.8125	190.5069	0.9986
**Met + DIP.1**	5.4624	0.9457	613.1538	604.3198	0.9958
**Met + DIP.2**	5.2850	0.9239	520.1887	515.9366	0.9966
**Met + DIP.3**	5.6141	0.9521	633.3944	629.8381	0.9955
**Met + NR.1**	3.8264	0.8335	480.2759	432.5906	0.9967
**Met + NR.2**	5.2562	0.9298	716.4000	728.0337	0.9945
**Met + NR.3**	3.8083	0.7695	700.9516	664.2721	0.9946

Table S3: Percentage of bacterial phyla in different treatment groups

Groups	Percentage of Bacterial Phyla									
	**Firmicutes**	**Bacteroidetes**	**Proteobacteria**	**Actinobacteria**	**TM7**	**Tenericutes**	**Deferribacteres**	**Verrucomicrobia**	**Cyanobacteria**	**Chloroflexi**
**Ctrl**	64.04	31.24	1.53	0.54	1.97	0.54	1.53	1.97	0.18	0
**Ctrl+DIP**	57.37	34.23	3.62	1.29	1.43	1.29	3.62	1.43	0.04	0
**Clin**	22.82	72.16	4.33	0.24	0	0.24	4.33	0	0	0
**Clin+DIP**	45.65	51.82	1.12	0.71	0.52	0.71	1.12	0.52	0	0
**Clin+NR**	55.21	39.94	3.98	0.4	0.22	0.4	3.98	0.22	0.01	0
**Met**	50.87	19.48	27.07	2.48	0	2.48	27.07	0	0	0
**Met+DIP**	41.95	54.89	1.54	1.17	0.26	1.17	1.54	0.26	0	0
**Met+NR**	54.87	18.2	25.09	0.67	0.64	0.67	25.07	0.64	0	0.09

Table S4: Percentage of bacterial family in different treatment groups

Groups	Percentage of Bacterial Family									
	**Lactobacillaceae**	**Bacteroidaceae**	**S24-7**	**Enterococcaceae**	**Lachnospiraceae**	**Enterobacteriaceae**	**Ruminococcaceae**	**Odoribacteraceae**	**Erysipelotrichaceae**	**Alcaligenaceae**
**Ctrl**	39.33	3.03	16.14	0.64	6.8	0.07	3.3	3	0.09	0.02
**Ctrl+DIP**	6.1	1.45	20.02	0.8	8.86	0.04	8.55	3.7	0.4	0
**Clin**	4.6	66.06	17.17	1.17	9.97	3.7	0.86	0.6	4.3	-
**Clin+DIP**	24.04	10.12	36.17	4.7	6.3	0.33	2.8	2	0.3	-
**Clin+NR**	29.68	33.45	4.18	1.94	14.73	3.3	1.6	0.5	2.2	-
**Met**	11.29	16.77	1.71	36.07	2.6	22.06	0.1	0	0.44	4.7
**Met+DIP**	26.39	10.68	42.24	0.44	3.78	0.06	3.4	0.3	0.08	0.2
**Met+NR**	16.27	2.62	11.64	30.14	1.94	20.75	1.3	0.5	0.59	3.4
